# HDS screening with patient-derived primary cells guided individualized therapy for esophageal squamous cell carcinoma–*in vivo* and *vitro*

**DOI:** 10.3389/fmed.2023.1212851

**Published:** 2023-08-02

**Authors:** Xing He, Hezhong Yan, Jie Hu, Xiaowei Duan, Mingjin Zhang, Haiqing Li, Jiaoxue Wang, Qian Gao, Senyuan Yu, Xilu Hou, Guobin Liao, Shicun Guo, Jin Li, Yurong Ge, Xiaolan Chen, Wenchao Wang, Jun Tang

**Affiliations:** ^1^Department of Gastroenterology, The 901th Hospital of Joint Logistics Support Force, Hefei, Anhui, China; ^2^Anhui Province Key Laboratory of Medical Physics and Technology, Institute of Health and Medical Technology, Hefei Institutes of Physical Science, Chinese Academy of Sciences, Hefei, Anhui, China

**Keywords:** esophageal squamous cell carcinoma, high-throughput drug sensitivity screening technology, drug sensitivity, patient-derived tumor xenografts, therapeutic efficacy

## Abstract

**Objective:**

To analyze and evaluate the role of the High-throughput Drug Sensitivity (HDS) screening strategy in identifying highly sensitive drugs against esophageal squamous cell carcinoma (ESCC).

**Methods:**

A total of 80 patients with progressive ESCC were randomly divided into the observation (40 cases) and the control groups (40 cases). In the observation group, primary ESCC cells were isolated from the tumor tissues with a gastroscope, and drug sensitivity screening was performed on cells derived from the 40 ESCC cases using the HDS method, followed by verification in a patient-derived tumor xenograft (PDX) mouse model. Finally, the differences in the therapeutic efficacy (levels of CEA, CYFRA21-1, SCCA after chemotherapy and the rates of overall survival, local progression, and distant metastasis at 12 months and 18 months time points after chemotherapy) were compared between the observation group (Screened drug-treated) and the control group (Paclitaxel combined with cisplatin regimen-treated).

**Results:**

Forty ESCC patients were screened for nine different high-sensitive chemotherapeutics, with the majority showing sensitivity to Bortezomib. Experiments on animal models revealed that the tumor tissue mass of PDX mice treated with the HDS-screened drug was significantly lower than that of the Paclitaxel-treated mice (*p* < 0.05), and the therapeutic efficacy of the observation group was better than the control group (*p* < 0.05).

**Conclusion:**

HDS screening technology can be beneficial in screening high-efficacy anticancer drugs for advanced-stage ESCC patients, thereby minimizing adverse drug toxicity in critically ill patients. Moreover, this study provides a new avenue for treating advanced ESCC patients with improved outcomes.

## Introduction

Esophageal carcinoma (EC) is a highly prevalent and dangerous malignancy of the gastrointestinal (GI) tract (1, 2), accounting for the seventh most common cancer worldwide as well as ranking sixth for cancer-related deaths (3). EC claims approximately 450,000 lives annually (4), with a 5-year survival rate of only 18% (5). Notably, the incidence of EC is increasing rapidly in several countries, including China, in recent times (6, 7). In China, EC causes about 150,000 annual deaths, which equals 21.8% of all cancer deaths, ranking fourth in the list of deadliest carcinomas. The etiology of EC is complex, many factors such as drinking, smoking, diet, genetics, microorganisms and gender have been reported to be associated with elevated risk of EC by previous studies (5–7). Therefore, it is technically challenging and difficult to reduce the occurrence of EC. EC patients primarily undergo endoscopic diagnosis for GI symptoms. Esophagectomy is the major surgical intervention in EC patients, while other treatment modalities involve chemo alone or a combination of chemo and radiotherapy, depending on the clinical characteristic of the cancerous lesion (8, 9). Most often, patients are diagnosed with an advanced stage EC, leaving no option for radical surgery. Importantly, postoperative recurrence and metastasis are frequently detected in surgical EC patients, hence, chemotherapy is considered a potentially effective second line of treatment (10). Interestingly, there is a wide variation in EC tumor classification in different geographical regions, such that >90% of EC cases in Western countries are adenocarcinomas, while esophageal squamous cell carcinoma (ESCC) is the most prevalent (>90% of all cases) among the Chinese populations (11). Therefore, this wide range of variabilities hinders the universal application of the National Comprehensive Cancer Network (NCCN) guidelines to Chinese ESCC patients, directly affecting their treatment outcomes and tumor recurrence. Moreover, establishing an *in vitro* screening strategy for determining patient-specific sensitivity of chemotherapeutics may resolve the discrepancies in the generalized application of NCCN guidelines.

**Figure 1:**
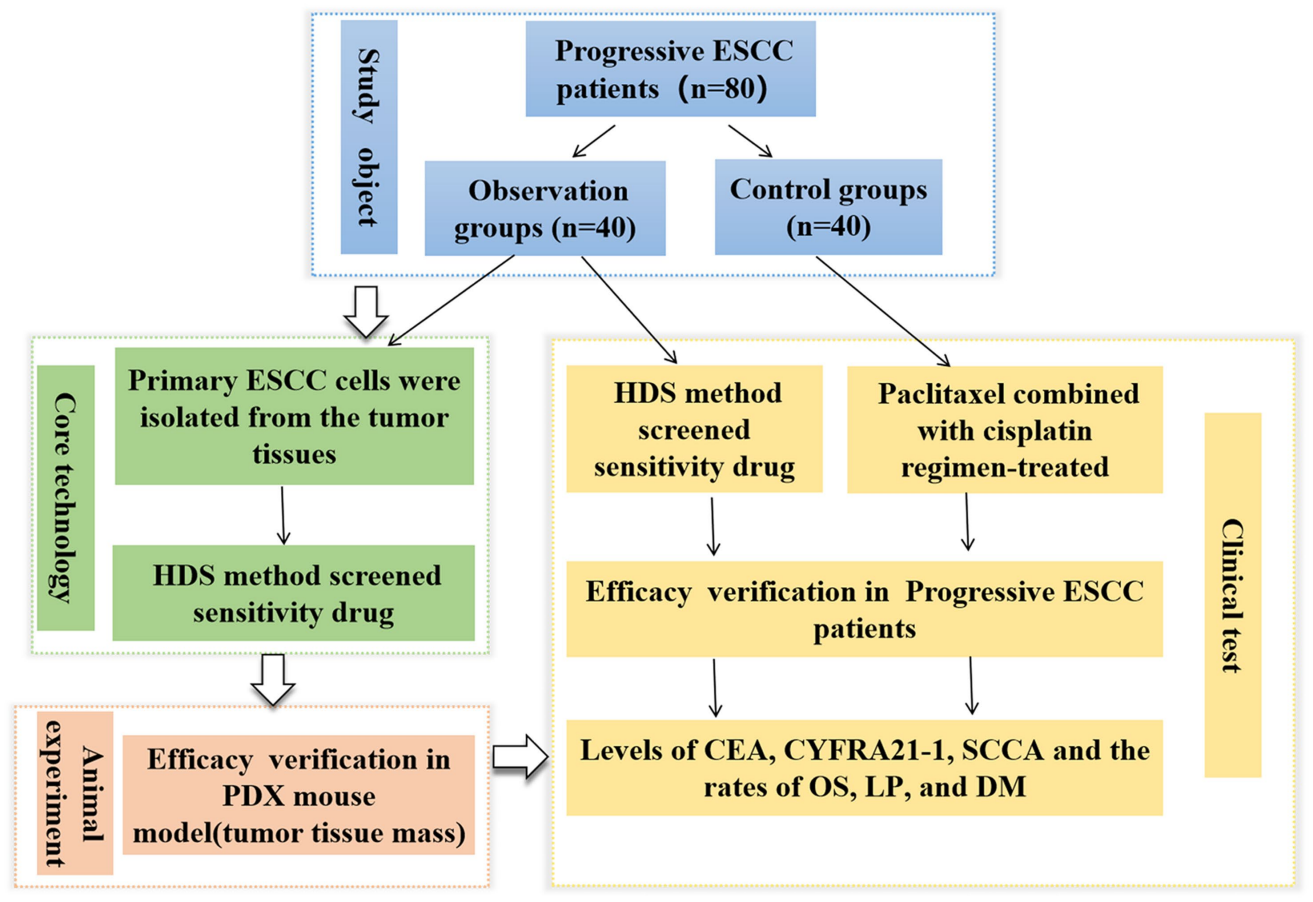
Flowchart showing the entire work procedure of this study including sections of study object, core technology, animal experiment and clinical test. ESCC, esophageal squamous cell carcinoma; PDX, patient-derived xenograft; HDS, High-throughput drug sensitivity.

High-throughput drug sensitivity (HDS) screening strategy is a new method with the advantages of high efficiency, wide coverage, and potential adaptation to personalized therapy, which are promising for determining the antitumor drug sensitivity in liver cancer, head and neck cancer, and lymphomas (12–14). However, the application of this technique in antitumor drug screening of ESCC is rarely reported. Therefore, this study aimed to generate ESCC patient-derived cell models for HDS analyses and develop a patient-derived xenograft (PDX) model of human ESCC in rodents for *in vivo* investigation. This study provides optimized assay platform able to offer drug screening outcomes in ESCC samples with varying clinical features and guides the precision therapy for ESCC patients (see Figure 1).

## Materials and methods

### Patient selection

A total of 80 outpatient and inpatient candidates with progressive ESCC were recruited from the Department of Gastroenterology of the 901 Hospital of the Joint Logistics and Security Forces of the Chinese People’s Liberation Army from December 2018 to December 2019 and randomly assigned to either the observation group or the control group (*n* = 40 per group). There were no statistical differences in terms of age distribution, male-to-female ratio, tumor characteristics, and patient’s Karnofsky score (15) between the two groups (*p* > 0.05). Patients’ inclusion criteria were: (1) compliance with the diagnostic criteria of progressive ESCC (16); (2) availability of complete clinical data, and (3) no indication for surgery. On the other hand, patients were excluded if they had: (1) cardiovascular diseases; (2) penetrating ulcers; (3) hepatic and renal insufficiency; (4) coagulation disorder, and (5) severe immune system diseases. The study was approved by the Institutional Ethics Committee (Ethics Committee of the 901th Hospital of the Joint Support Force), and all participants provided their signed informed consent by themselves or their family members (Chinese clinical trial registration number: ChiCTR2300068566) (see Table 1).

**Table 1:** Comparison of general information between the two groups of patients.

Projects	Observation group	Control group	*t*/*χ*^2^	*p*-value
Age(y)	55.15 ± 6.25	54.85 ± 6.12	0.217	0.829
Gender			0.091	0.762
Male	33 (82.50%)	34 (85.00%)		
Female	7 (17.50%)	6 (15.00%)		
Karnofsky score	70.28 ± 3.11	70.68 ± 3.17	0.570	0.571
Histological grading			0.315	0.854
Low differentiation	12 (30.00%)	10 (25.00%)		
Medium-high divergence	14 (35.00%)	14 (35.00%)		
Uncertain	14 (35.00%)	16 (40.00%)		
Distant metastasis rate			0.457	0.499
Transfers	16 (40.00%)	19 (47.50%)		
Not transferred	24 (60.00%)	21 (52.50%)		
Tumor diameter (cm)	4.65 ± 1.03	4.85 ± 1.18	0.808	0.422

### Experimental animals

Severely immunodeficient (NCG) six-week-old female mice were purchased from Nanjing Model Animal Research Institute. The animal experiment protocol was approved by the Institutional Animal Care and Use Committee (Ethics Committee of the 901th Hospital of the Joint Support Force).

The main instruments and reagents that were used in this study are listed in Table 2.

**Table 2:** Main experimental equipment and reagents used in the experiment.

Name	Manufacturers	Model/Specification
Flow Image Counter	Jiangsu Zhuo Wei Technology Co.	JIMBIO FILE
Electron Microscope	Invitrogen Corporation	EVOS M500
384-well cell culture plate	Perkin Elmer Corporation	608,598
High throughput automation workstation	Perkin Elmer Corporation	JANUS
Enzyme Labels	Perkin Elmer Corporation	Envision
0.05% trypsin	Gibco Corporation	25,300,062
0.25% trypsin	Corning Incorporated	25-053-CI
Trypan blue staining solution	Biosharp Corporation	BL707A
Erythrocyte lysate	Sigma Corporation	R7757-100ML
Fetal bovine serum	EXCELL Corporation	FND500
Cell Titer-Glo Assay	Promega Corporation	DD1101

### Experimental methods

**Figure 2:**
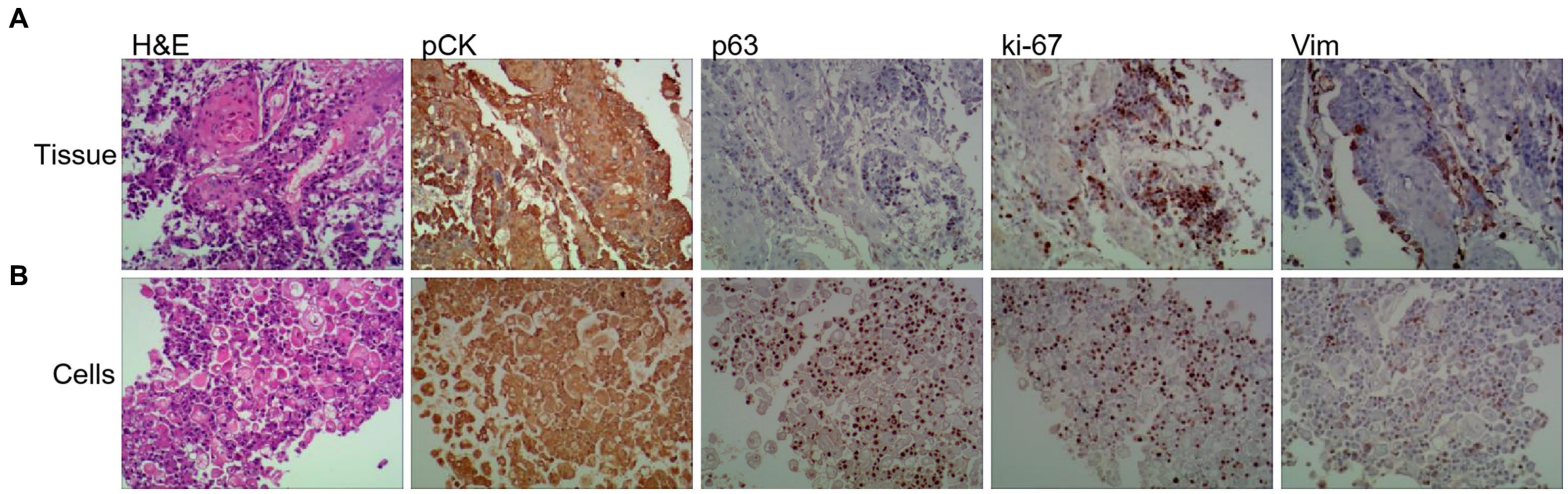
Tumor primary cell cultures share histopathological features with patient-matched tissue and disruption of polarity. Representative images of H&E, and IHC of Vim (Vimentin), panCK (pan-cytokeratin), ki-67 and p63 from primary tissue **(A)** and patient-matched primary cell **(B)** (20× magnifification used for primary cells and tissues).

**Figure 3:**
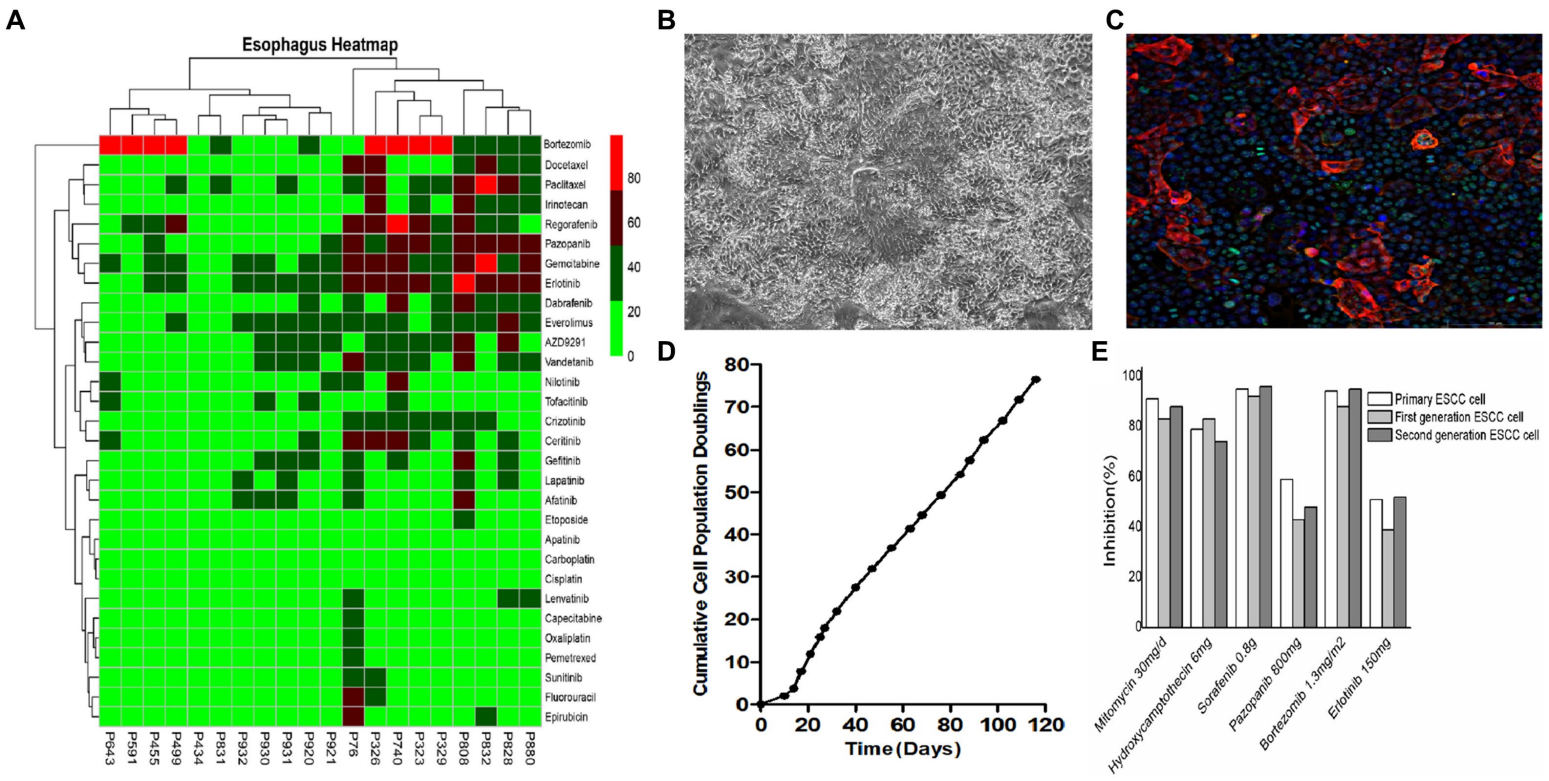
*In vitro* culture and drug sensitivity testing in ESCC cells. **(A)** Primary cells derived from different ESCC patients exhibited different sensitivities to different drugs. **(B)** Representative bright field images of primary ESCC cells in culture. **(C)** Primary ESCC cells characterized by immunofluorescence (IF) staining with squamous cell carcinoma marker anti-p63 antibody. **(D)** Cell proliferation curve of ESCC cells. **(E)** There was no significant difference in drug susceptibility between algebraic ESCC primary cells amplified *in vitro*.

ESCC primary cell lines were derived from biopsied patient tumor tissue specimens and subsequently characterized as follows:

1. Gastroscopy and biopsy of ESCC tumors were performed using an Olympus CLV-260 electronic gastroscope. Hematoxylin and eosin (H&E) staining and immunohistochemical (IHC) analysis using anti-pCK, anti-p63, anti-Ki-67, and anti-Vim antibodies indicated that tumor tissues contained abundant ESCC cells (Figure 2A).
2. Isolation of ESCC primary cells was performed in the following steps: tissue washing, digestion, centrifugation, collection and resuspension of the cell pellet in the basal medium, centrifugation, removal of the supernatant and lysis of blood cells in appropriate buffer, and final collection of ESCC primary cells by centrifugation.
3. Culture of tumor-derived ESCC cells for (1) microscopic studies, (2) live cell counting, and (3) cell culture for downstream assays (Figure 3B).
4. Identification and characterization of cultured ESCC primary cells (Figures 3D,E).
5. The cells were washed with 200 μL of 0.25% trypsin for 1 min, aspirated, and then 500 μL of 0.05% trypsin solution was added to each well and placed in an incubator at 37°C with 5% CO_2_ for 10 min until complete cell detachment was observed under the microscope. After centrifugation at 1500 rpm for 4 min, the supernatant was discarded, and 500 μL of ESCC primary cell medium was added to resuspend and plate the cells (Figure 2B and Figure 3C).

### HDS screening for drug sensitivity in patient-derived ESCC lines

The patient-specific primary ESCC cells were separately treated with different first-line and second-line drug regimens as well as subjected to the FDA drug library screening using the HDS method. Steps in this process include:

1. Preparation of cultured cells on petri dishes: ESCC primary cells were harvested by trypsin-digestion, centrifuged, and resuspended in DPBS buffer for cell counting by an image cytometer. The cell suspension was then diluted to 6 × 10^4^–1 × 10^5^ cells/ml, and seeded into 384-well plates using 50 μL of cell suspension per well.
2. Serial dilutions of drugs: the testing drug was serially diluted to different working concentrations (10 μM, 3 μM, 1 μM, 0.3 μM, 0.1 μM, 0.03 μM, and 0.01 μM).
3. Drug treatments: 24 h after seeding, cells were treated with different doses of serially diluted drug solutions or vehicle control in triplicates in 384-well plates using the automated JANUS liquid handler workstation.
4. Cell viability testing: 72 h post-treatment, the cell viability in different treatment and control groups was measured using a plate reader-based CellTiter Glo assay and calculated by the formula, Cell viability (%) = luminescence value of the spiked wells/luminescence value of the control wells × 100%. Cell inhibition (%) = 100- Cell viability (Figure 3A and Table 3).

**Table 3:** The response rate of ESCC cells by different therapeutic drugs determined by HDS technique.

Drug	Dose	Response rate (%)	Drug	Dose	Response rate (%)
Paclitaxel	200 mg/m^2^	4/40 (10)	Doxorubicin	60 mg/m^2^	10/40 (25)
Carboplatin	AUC5	0/40 (0)	Gemcitabine	1000 mg/m^2^	7/40 (17.5)
Cisplatin	100 mg/m^2^	0/40 (0)	Hydroxycamptothecine	6 mg	16/40 (40)
Fluorouracil	2600 mg/m^2^	0/40 (0)	Vincristine	1.4 mg/m^2^	0/40 (0)
Oxaliplatin	130 mg/m^2^	1/40 (2.5)	Etoposide	100 mg/m^2^	1/40 (2.5)
Capecitabine	1000 mg/m^2^	1/40 (2.5)	Cytarabine	2 mg/kg	2/40 (5)
Epirubicin	50 mg/m^2^	0/40 (0)	Pirarubicin	40 mg/m^2^	3/40 (7.5)
Irinotecan	300 mg/m^2^	3/40 (7.5)	Everolimus	10 mg	0/40 (0)
Docetaxel	75 mg/m^2^	2/40 (5)	Axitinib	10 mg PO	1/40 (2.5)
Tegafur	80 mg/m^2^	0/40 (0)	Regorafenib	160 mg PO	3/40 (7.5)
Mitomycin	30 mg/d	4/40 (10)	Sorafenib	0.8 g PO	11/40 (27.5)
Sunitinib	50 mg PO	0/40 (0)	Gefitinib	250 mg PO	2/40 (5)
Apatinib	850 mg	1/40 (2.5)	Erlotinib	150 mg PO	1/40 (2.5)
Pemetrexed	500 mg/m^2^	4/40 (10)	Osimertinib	80 mg PO	2/40 (5)
Raltitrexed	3 mg/m^2^	0/40 (0)	Vandetanib	300 mg PO	1/40 (2.5)
Lapatinib	1250 mg PO	0/40 (0)	Lenvatinib	24 mg PO	1/40 (2.5)
Etoposide	200 mg/m^2^	1/40 (2.5)	Pazopanib	800 mg PO	3/40 (7.5)
Icotinib	125 mg PO	0/40 (0)	Nintedanib	100 mg	4/40 (10)
Dabrafenib	150 mg PO	1/40 (2.5)	Afatinib	40 mg PO	0/40 (0)
Ceritinib	750 mg PO	2/40 (5)	Crizotinib	250 mg PO	1/40 (2.5)
Dasatinib	140 mg PO	3/40 (7.5)	Abiraterone	1 g PO	1/40 (2.5)
Imatinib	400 mg PO	5/40 (12.5)	Bortezomib	1.3 mg/m^2^	30/40 (75)
Ginsenoside Rh2	TCM Monomer	2/40 (5)	Tripterine	TCM	1/40 (2.5)
Carmustine	100 mg/m^2^	0/40 (0)	Lomidoxin	400 mg PO	1/40 (2.5)

TCM, traditional Chinese medicine; PO, eros; Response rate **=** the number of patients with inhibition rate > 50% among total patients (percentage rate of response patient number/total number of people × 100%).

### *In vivo* validation of drug effects in PDX mouse model

#### Model construction

Approximately 5 × 10^6^ ESCC progenitor cells were injected into the ESCC fat pad and the axillary area of the right forelimb of 6-week-old female NCG mice. The volume and growth rate of ESCC tumors in mice were routinely recorded every 3 days. Tumor formation was observed at both graft sites on day 15 post-administration. Tumor proliferation was evident from day 15 to day 30, indicating a successful model establishment.

#### Dose optimization

The doses of the screening drugs were set at 25, 50, and 100 mg/kg/d. After the model was successfully constructed, the drug treatment group mice were administered with designated drug regimens either by oral gavage or intraperitoneal (i.p) injection for 20 days, while paclitaxel (70 mg/kg/d) (17) treated mice served as the positive control group, and vehicle (DMSO)-treated mice were included in the sham group.

### Treatment protocol for ESCC subjects in the observation and control groups

Forty ESCC patients in the control group were treated with paclitaxel combined with a cisplatin regimen (18). On day 1, 135 mg/m^2^ of paclitaxel injection (Jiangsu Aosikang Pharma., State Drug Quantification H20083848; 2 mL: 30 mg) was added to 500 mL of glucose injection (Jiangsu Hengrui Pharma., State Drug Quantification H32022368; 250 mL: 12.5 g) and diluted into intravenous (i.v) drip for 3 h/d. On days 2–4, subjects received i.v injections of 50 mg/m^2^ of cisplatin (Dezhou Deyao Pharma., State Drug Quantification H20023236; 20 mg), dissolved in 500 mL of 0.9% injection-grade sodium chloride solution (Shijiazhuang IV Pharma., State Drug Quantification H13023200; 500 mL) for 2 h each time in a stretch of 3 weeks. The treatment lasted six cycles.

On the other hand, 40 ESCC patients in the observation group were treated according to screened therapeutic agents.

We compared several parameters between these two groups, such as the sensitivity of different anticancer drugs in ESCC cells by the HDS method, the difference in tumor growth curves before and after drug therapy in the PDX mice as well as relative expression levels of tumor markers, including carcinoembryonic antigen (CEA), serum cytokeratin protein fragment 19 (CYFRA21-1) and squamous epithelial cell carcinoma antigen (SCCA) in the ESCC patients. Furthermore, we compared the differences in overall survival (OS) rates, local progression (LP) rates, and distant metastasis (DM) rates between the two groups of patients at 12 and 18 months after chemotherapy. OS rate = number of survivors/total number × 100%. LP rate = number of local tumor diameter increase/total number × 100%. DM rate = number of new distant metastases/total number × 100%.

### Statistical analysis

SPSS v22.0 software (IBM Corp., Armonk, NY, United States) was used for all statistical analyses. Measured and counted data were expressed as xs and %, respectively. The t-test was used to compare the differences in expressions of CEA, CYFRA21-1, and SCCA in ESSC patients before and after treatment with different drugs as well as tumor volumes and masses in PDX models under various treatment conditions. Least significant difference (LSD) method was used for multiple comparisons. Chi-square (*χ*^2^) test was used to compare the rate of local progression and distant metastasis between the two groups. A *p*-value of less than 0.05 was considered statistically significant.

## Results

### Identification of highly effective drugs against patient-derived ESCC cell models by HDS method

Forty ESCC patients were subjected to treatment with 9 different highly sensitive chemotherapeutic agents, with the largest number of patients exhibiting significant sensitivities to bortezomib, as described in Table 4.

**Table 4:** Screening of drug sensitivity by measuring tumor cell inhibition rates (%)in 40 ESCC patients in the observation group.

No.	Screening drugs	Inhibition rate (%)	No.	Screening drugs	Inhibition rate (%)	No.	Screening drugs	Inhibition rate (%)	No.	Screening drugs	Inhibition rate (%)
1	Imatinib	98.1	11	Adriamycin	88.93	21	Bortezomib	53.23	31	Bortezomib	53.23
2	Bortezomib	53.23	12	Bortezomib	79.37	22	Sorafenib	96.9	32	Everolimus	36.84
3	Gemcitabine	48.81	13	HCPT	33.39	23	Bortezomib	88.38	33	Bortezomib	91.01
4	Sorafenib	98.65	14	Sorafenib	95.70	24	Bortezomib	75.09	34	Bortezomib	91.01
5	Tripterine	91.90	15	Bortezomib	91.56	25	Bortezomib	53.23	35	Bortezomib	53.23
6	Bortezomib	88.38	16	Bortezomib	95.04	26	Tripterine	17.78	36	Lomidoxin	96.51
7	Bortezomib	88.38	17	Bortezomib	88.38	27	Bortezomib	91.05	37	Bortezomib	89.39
8	Everolimus	36.84	18	Bortezomib	83.67	28	Bortezomib	53.23	38	Sorafenib	95.97
9	Bortezomib	88.38	19	Sorafenib	98.2	29	Bortezomib	53.23	39	HCPT	95.799
10	HCPT	61.09	20	Bortezomib	91.48	30	Bortezomib	53.23	40	Adriamycin	96.34

HCPT, Hydroxycamptothecin.

### HDS-screened drugs show inhibition of tumor growth in the PDX mice

**Figure 4:**
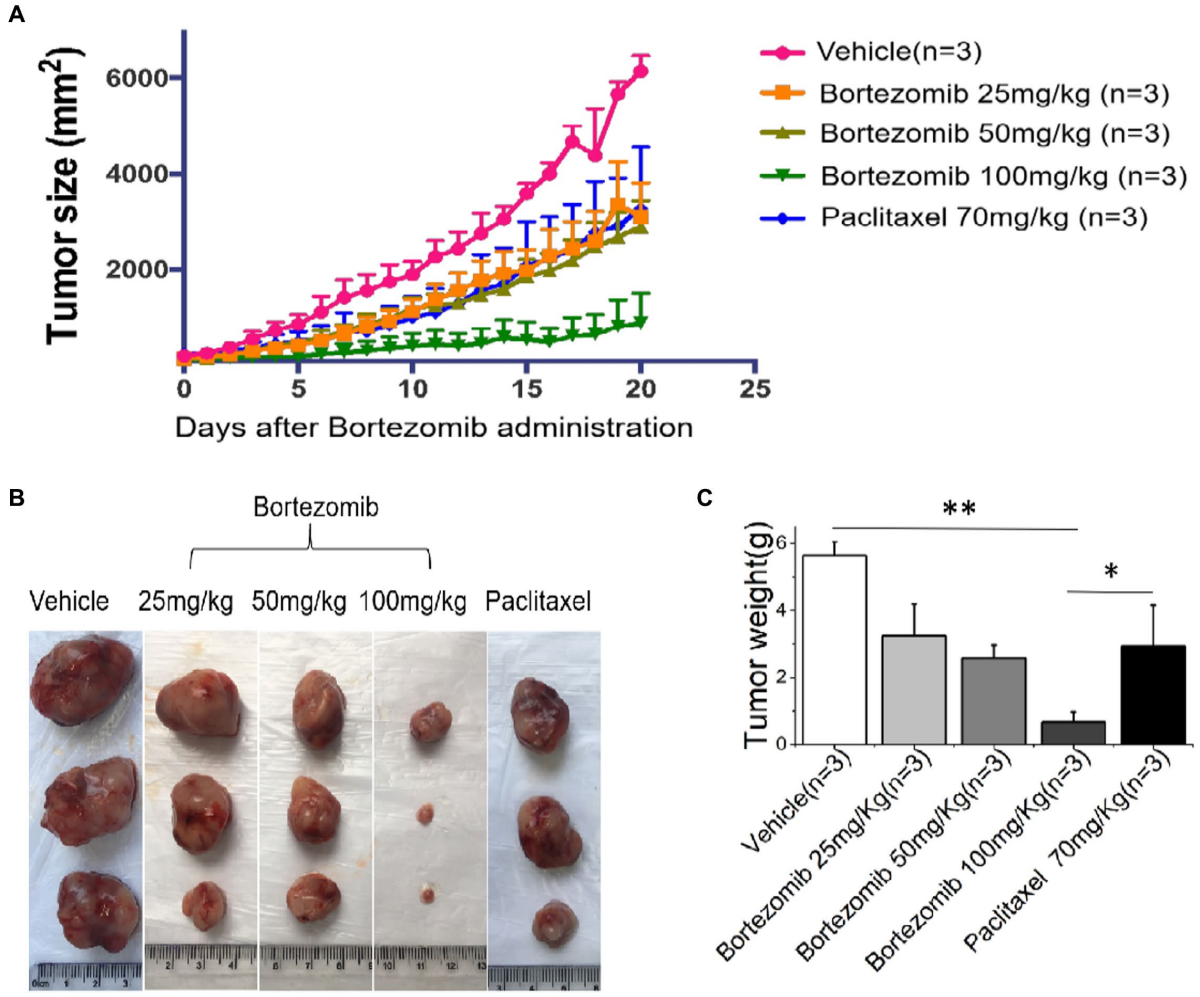
An improved inhibitory effect of HDS-screened drugs on tumor cell growth in PDX animal models compared to paclitaxel and vehicle. **(A)** The PDX mice were treated with vehicle, Bortezomib (25 mg/kg, 50 mg/kg, 100 mg/kg), or Paclitaxel (70 mg/kg) every day for 20 consecutive days. Tumor volumes were measured each day. The curves show the mean volume ± SD of tumor sizes. **(B)** Representative images of xenografts harvested after 20 days of treatment for comparison of tumor volumes across the groups. **(C)** The bar chart shows the difference in xenograft quality in different groups.^*^p < 0.05, ^**^p < 0.01.

We found that HDS-screened drug-treated PDX mice had significantly smaller tumor Volumes and masses compared to that in the vehicle-treated sham (*p* < 0.05), and Paclitaxel treatment groups, as illustrated in Figure 4.

### Treatment with antitumor agents alleviates the expression of tumor markers in ESCC patients

There were no statistical differences in the expression of CEA, CYFRA21-1, and SCCA between the two groups at the baseline (t = 0.327, 0.088, 0.276; *p* = 0.745, 0.930, 0.783), while significantly lower expression levels of these protein markers were detected in the observation group than those in the control group (t = 5.692, 5.748, 10.817; *p* < 0.001) after chemotherapy, as shown in Table 5.

**Table 5:** Comparison of tumor markers’ levels before and after drug therapy between the observation and control groups.

Group	Time	CEA (ng/mL)	CYFRA21-1 (ng/mL)	SCC (μg/L)
Observation group	Before treatment	9.17 ± 3.14	4.52 ± 1.61	2.33 ± 1.01
	After treatment	2.37 ± 1.05^ab^	2.11 ± 1.09^ab^	0.76 ± 0.28^ab^
Control group	Before treatment	8.94 ± 3.16	4.49 ± 1.45	2.27 ± 0.93
	After treatment	4.13 ± 1.65^a^	3.57 ± 1.18^a^	1.54 ± 0.36^a^

^a^*p* < 0.05, compared with the control group before treatment. ^b^*p* < 0.05, compared with the control group after treatment. CEA, carcinoembryonic antigen, CYFRA21-1, serum cytokeratin protein fragment 19; SCCA, squamous epithelial cell carcinoma antigen.

### HDS-guided treatment enhanced the clinical efficacy of chemotherapeutics

We found that the OS rates at 12 months and 18 months were significantly higher in the observation group than those in the control group (*p* < 0.05), while rates of LP and DM at 18 months were significantly lower in the observation group than those in the control group (*p* < 0.05), as shown in Table 6.

**Table 6:** Comparison of treatment results between the observation and control groups (*n*, %).

Group	12-month OS rates	18-month OS rates	LP rates	DM rates
Observation group	31 (61.5)	23 (42.7)	11 (27.5)	12 (30)
Control group	22 (49.8)	14 (29.5)	21 (70)	21 (70)
*χ*2 value	4.528	4.073	5.208	4.178
*p* value	0.033	0.044	0.023	0.041

OS, overall survival; LP, local progression; DM, distant metastasis.

## Discussion

Since the majority of patients with progressive ESCC are diagnosed at the advanced stage, most clinicians adopt palliative radiotherapy, chemotherapy, biological therapy, and traditional Chinese medicine (TCM) therapies (19–22). Notably, due to extensive heterogeneity in tumor characteristics across ESCC patients, individualized chemotherapeutic strategies are urgently needed to achieve the best therapeutic outcome and minimum drug toxicity (23, 24). Most clinical researchers exploit the patient-derived tumor cell-derived PDX rodents to assess the cell growth inhibitory effect of different regimens of anticancer drugs to overcome the problem of adverse drug toxicity in critically ill ESCC patients (25, 26). However, these *in vitro* testing models have their limitations, such as high cost and limited capability in accommodating large panels of therapeutic drugs. Some studies have demonstrated the broader benefits of organoid models using patient-derived cancer stem cells mimicking *in vivo* tumor microenvironment (TME) for drug screening (27, 28). Again, the efficacy of this model is limited by challenges in accurately modeling cancer organoids, high maintenance costs, and difficulty in simultaneously generating numerous organoids for high-throughput screening strategies.

Recently, high-throughput gene sequencing (HGS) technology has been used for the targeted therapy of ESCC. Some groups have reported the genetic landscape of human ESCCs using the whole genome and exome sequencing approaches (29–31), which has led to promising results in early studies of personalized and targeted therapies (32). However, due to an increase in the oncogenic mutational burden, differences in cellular signaling pathways, and complex interactions among tumor genes, the same tumor gene target may respond differentially to different drugs affecting the overall outcome of individualized therapy (33).

In contrast, the HDS technology used by our group extended the scope of screening with a large panel of FDA-approved drugs for determining the best possible drug regimen exerting maximum cell growth inhibitory effects. With this technically upgraded screening platform, we can rapidly identify highly sensitive and targeted chemotherapeutics for different ESCC patients, thereby solving most of the previously mentioned technical hurdles.

The primary aim of this study was to obtain high-quality primary tumor cells from ESCC patients and subsequently exploit them for *in vitro* drug sensitivity screening strategies as an alternative to *in vivo* models. We used fresh tumor tissue masses to isolate primary ESCC cells through trypsin digestion with a success rate as high as 90%, which was considerably higher than that reported in other studies (34). The HDS technique developed by our group could simultaneously measure the sensitivity of more than 300 drugs to patient-derived ESCC tumor cells, along with the screening of optimal drug concentrations to reduce the adverse drug toxicity effects. In this study, primary tumor cells from 40 ESCC patients were tested for drug sensitivity against 9 different chemotherapeutic agents, which fully illustrated the inter-patient heterogeneity of tumor cells (35). Moreover, the results of this study further signify the importance of individualized chemotherapeutic regimens in treating highly heterogeneous carcinomas like ESCC. Through this screening assay, we noticed that most ESCC patients were sensitive to Bortezomib.

Bortezomib is a 20S proteasome inhibitor that exerts its antitumor effects by interfering with cell signaling, causing cell cycle arrest, inducing apoptosis, and inhibiting angiogenesis and is currently used mainly for the treatment of multiple myeloma (36, 37). However, one study (38) reported its role in enhancing the sensitivity of ESCC to radiotherapy. Besides, there are few a studies on the application of Bortezomib in chemotherapy for advanced-stage ESCC (39). Here, we found that Bortezomib was effective in chemotherapy for esophageal squamous carcinoma by *in vitro* HDS assay, which might provide a new avenue for the treatment of ESCC.

To further verify the results of *in vitro* experiments, the previously reported PDX model (40) was used to determine the therapeutic effects of screened anticancer drugs *in vivo*. We exploited patient-derived primary ESCC cells for constructing the PDX mouse model to screen potential anticancer drugs. In addition, we used Paclitaxel (41) as the positive control in this animal study. The results showed that the tumor tissue mass and volume of PDX mice treated with screened high-sensitivity drugs were significantly smaller than those treated with Paclitaxel or the vehicle control (*p* < 0.05). Finally, we determined the optimal drug concentration to achieve the best possible tumor growth inhibition in this PDX mouse model.

After achieving encouraging results in animal experiments, we conducted individualized treatment for 40 ESCC patients with Bortezomib and compared the anticancer efficacy with 40 ESCC patients who received Paclitaxel combined with cisplatin chemotherapy in the same period of time. We first analyzed the differences in expressions of tumor markers CEA, CYFRA21-1, and SCCA between the two groups. CEA is one of the first tumor markers found to be widely present in tumor cells. CYFRA21-1 is also commonly detected in lung and esophageal tumor cells and exhibits a high sensitivity along with the diagnostic value in the evaluation of therapeutic effect against ESCC (42). SCCA is mainly present in tumor cells of the lung, pharynx, and esophagus and exhibits a high sensitivity for occurrence and progression of ESCC with high specificity (43). Some studies (44–46) have reported that the combination of these three biomarkers could be a valuable prediction tool for the precise diagnosis of EC and for predicting the disease progression rate as well. Here, we found that CEA, CYFRA21-1, and SCCA levels were significantly lower than those in the control group after chemotherapy treatment in the observation group (*p* < 0.05), suggesting that the HDS-screened anticancer drug was effective in controlling the ESCC tumor growth in the observation group. Furthermore, we analyzed the treatment efficacies in the two groups based on clinical symptoms and found that the 12-month and 18-month OS rates in the observation group were significantly higher than those in the control group (*p* < 0.05), while rates of LP and DM were significantly lower than those in the control group (*p* < 0.05), indicating promising efficacy of HDS-screened drugs against ESCC.

In summary, the HDS method is an efficient, reproducible, and easy-to-use drug sensitivity screening tool that can expedite the ESCC diagnosis, especially for terminally ill patients. Moreover, drugs for antitumor sensitivity on the HDS platform consistently exhibited significant inhibitory effects on ESCC tumor growth in disease animal models as well as human ESCC patients.

## Data availability statement

The original contributions presented in the study are included in the article/Supplementary materials, further inquiries can be directed to the corresponding authors.

## Ethics statement

The studies involving human participants were reviewed and approved by Ethics Committee of the 901th Hospital of the Joint Support Force. The patients/participants provided their written informed consent to participate in this study. The animal study was reviewed and approved by Ethics Committee of the 901th Hospital of the Joint Support Force.

## Author contributions

The study was conceived and designed by XnH, JT, HY, JH, and WW. XnH, HY, and JH equally contributed to performing all the required experiments, analyzing data, preparing the manuscript, and further revisions. SY and XD contributed to the data acquisition and analysis. JT, HL, JW, JL, YG, XC, and MZ provided human adipose tissue samples. JH, HY, WW, and XnH performed the HDS experiments. QG, XlH, GL, and JH performed animal studies and immunohistochemical analysis. XlH, HY, XD, HL, JW, SG, and JT were responsible for clinical studies. WW and JT provided critical suggestions for this manuscript. All authors contributed to the article and approved the submitted version.

## Funding

This study was supported by the Anhui Key Research and Development Program (1804 h08020257).

## Conflict of interest

The authors declare that the research was conducted in the absence of any commercial or financial relationships that could be construed as a potential conflict of interest.

## Publisher’s note

All claims expressed in this article are solely those of the authors and do not necessarily represent those of their affiliated organizations, or those of the publisher, the editors and the reviewers. Any product that may be evaluated in this article, or claim that may be made by its manufacturer, is not guaranteed or endorsed by the publisher.

## Abbreviations

HDS: High-throughput Drug Sensitivity
EC: Esophageal Carcinoma
ESCC: Esophageal Squamous Cell Carcinoma
PDX: Patient-derived Tumor Xenograft
OS: Overall Survival
LP: Local Progression
DM: Distant Metastasis
CEA: Carcinoembryonic Antigen
CYFRA21-1: Serum Cytokeratin Protein Fragment 19
SCCA: Squamous Epithelial Cell Carcinoma Antigen
NCCN: National Comprehensive Cancer Network
FDA: Food and Drug Administration
HGS: High-throughput Gene Sequencing
