# Supplementary figures and images for: HDS screening with patient-derived primary cells guided individualized therapy for esophageal squamous cell carcinoma–in vivo and vitro
Source: Front Med (Lausanne). 2023 Aug 2;10:1212851. doi: 10.3389/fmed.2023.1212851 (PMC10433228; doi:10.3389/fmed.2023.1212851)

**Supplement 2:**

**H&E**

**P63**

**ki-67**

**pCK**


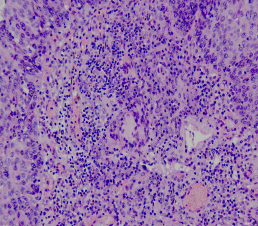

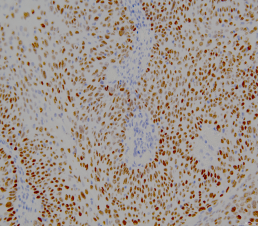

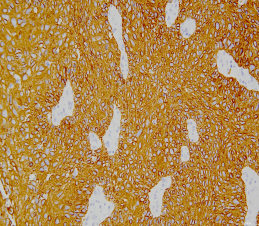

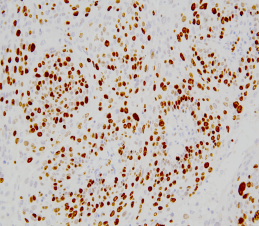

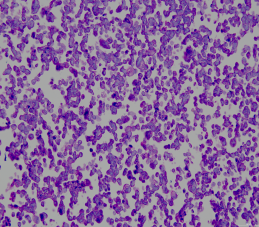

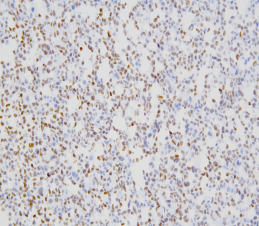

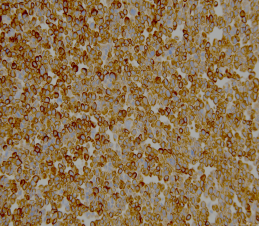

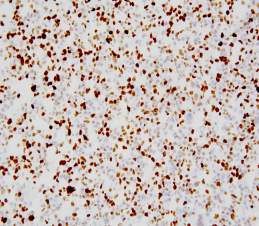


**Tissue**

**Cell**


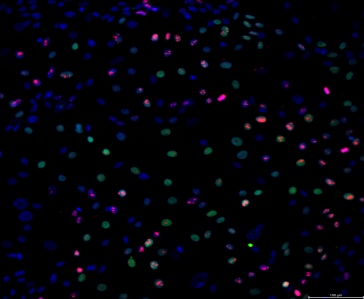
**Supplement 3:**

**P63 ki-67**

Supplement: Supplementary file 2 [file Data_Sheet_2.docx]
